# Ion-Triggered In Situ Gelling Nanoemulgel as a Platform for Nose-to-Brain Delivery of Small Lipophilic Molecules

**DOI:** 10.3390/pharmaceutics13081216

**Published:** 2021-08-06

**Authors:** Sreeharsha Nagaraja, Girish Meravanige Basavarajappa, Ranjith Kumar Karnati, Esam Mohamed Bakir, Swati Pund

**Affiliations:** 1 Department of Pharmaceutical Sciences, College of Clinical Pharmacy, King Faisal University, Al-Ahsa 31982, Saudi Arabia; 2 Department of Pharmaceutics, Vidya Siri College of Pharmacy, Off Sarjapura Road, Bangalore 560035, India; 3 Department of Biomedical Sciences, College of Medicine, King Faisal University, Al-Ahsa 31982, Saudi Arabia; gmeravanige@kfu.edu.sa; 4 Department of Chemistry, College of Science, King Faisal University, Al-Ahsa 31982, Saudi Arabia; rkarnati@kfu.edu.sa (R.K.K.); ebakir@kfu.edu.sa (E.M.B.); 5 Nanomedicine Laboratory, Department of Biosciences and Bioengineering, Indian Institute of Technology-Bombay, Mumbai 400076, India; swatipund@gmail.com or

**Keywords:** self-emulsifying, naringin, ex vivo diffusion, biocompatibility, in situ gel, nanoemulgel

## Abstract

**Background:** Intranasal route offers a direct nose-to-brain delivery via olfactory and trigeminal nerves and minimizes the systemic exposure of the drug. Although reliable and non-invasive, intranasal administration of lipophilic neuroprotective agents for brain targeting is still challenging. Literature focuses on naturally-derived compounds as a promising therapeutics for chronic brain diseases. Naringin, a natural flavonoid obtained from citrus fruits possesses neuroprotective effects. By regulating multiple crucial cellular signaling pathways, naringin acts on several therapeutic targets that make it suitable for the treatment of neurodegenerative diseases like Alzheimer’s disease and making it a suitable candidate for nasal administration. However, the hydrophobicity of naringin is the primary challenge to formulate it in an aqueous system for nasal administration. **Method:** We designed a lipid-based nanoemulsifying drug delivery system of naringin using Acrysol K140 as an oil, Tween 80 as a surfactant and Transcutol HP as a cosolvent, to improve solubility and harness the benefits of nanosizing like improved cellular penetration. Intranasal instillations of therapeutic agents have limited efficacy due to drug washout and inadequate adherence to the nasal mucosa. Therefore, we reconstituted the naringin self-emulsifying system in a smart, biodegradable, ion-triggered in situ gelling hydrogel and optimized for desirable gel characteristics. The naringin-loaded composition was optimized and characterized for various physicochemical and rheological properties. **Results:** The formulation showed a mean droplet size 152.03 ± 4.6 nm with a polydispersity index <0.23. Ex vivo transmucosal permeation kinetics of the developed formulation through sheep nasal mucosa showed sustained diffusion and enhanced steady-state flux and permeability coefficient. Scanning and transmission electron microscopy revealed the spherical shape of emulsion droplets and entrapment of droplets in a gel structure. The formulation showed excellent biocompatibility as analyzed from the viability of L929 fibroblast cells and nasal mucosa histopathology after treatment. In vivo biodistribution studies revealed significantly higher drug transport and brain targeting efficiency. **Conclusion:** In situ gelling system with nanoemulsified naringin demonstrated a safe nasal delivery providing a new dimension to the treatment of chronic neurodegenerative diseases using small hydrophobic phytoconstituents with minimization of dose and related systemic adverse effects.

## 1. Introduction

The central nervous system (CNS) is a complex, but a sophisticated system that regulates and coordinates several body activities. It is vulnerable to vascular, structural, degeneration and functional disorders [1]. Thus, CNS disorders represent increasing social and economic problems all over the world due to high morbidity and mortality [2]. Furthermore, many CNS diseases like Alzheimer’s disease and Parkinson’s disease need chronic therapies [3]. Drug delivery to the brain depicts a major challenge as many of the drugs do not cross the blood-brain barrier (BBB) [4]. Many strategies have been explored for effective drug delivery to the brain by vanquishing the challenges of BBB, both invasively and non-invasively [4]. BBB is highly restrictive in the access of molecules to the brain; it allows entry of only nutrients and mediators that are required to maintain brain homeostasis [5]. Generally, low molecular weight compounds (<400 Da) which are lipophilic and non-ionizable at physiological pH can cross BBB through diffusion [6]. However, targeting the drugs to the brain still remains a challenge. Invasive approaches are a non-preferred method of drug delivery to the brain [7]. The intranasal route of administration is easily amenable to self-administration and offers a non-invasive and virtually painless alternative to oral and parenteral administration for the delivery of drugs to the CNS [8]. In addition, it has been found that there is a direct connection between the olfactory and trigeminal regions through the sub-mucosa space of the nose into the cerebrospinal fluid compartment of the brain [9]. Thus, intranasal drug delivery can effectively deliver the drug to CNS by circumventing the BBB. Several studies have demonstrated the potential of naturally-derived bioactive phytoconstituents for the treatment of CNS disorders. Phytochemicals have the advantages of abundant resources, good curative effects and little or no side effects [10]. Naringin, a polyphenolic bioflavonoid possesses pharmacological effects such as anti-inflammatory, anti-cancer, antioxidant and neuroprotective effects [11]. There is increasing evidence on its variety of therapeutic targets along with complex signaling pathways, which suggest naringin a possible therapeutic candidate in several neurodegenerative diseases [12,13,14]. However, naringin is poorly water soluble which hinders further studies on its pharmacological applications [15]. Out of several methods to improve solubility [16,17,18], self-emulsifying systems offers an easily scalable strategy to overcome the poor bioavailability as well as permeability issues [18,19,20]. Self-emulsifying can effectively target drugs to CNS because of their particle size, high drug loading capacity, lipophilicity and relatively high physical stability. The surfactant used in such formulations increases membrane permeability and the oils enhance lymphatic absorption. In addition, for nose-to-brain delivery, particle size and lipophilicity are essential characteristics to be presented by the delivery system [21,22].

However, liquid formulations for intranasal administration are not favorable as rapid naso-mucociliary clearance shortens the residence time of the liquid formulation that eventually results in poor bioavailability. Utilization of in situ gelling systems wherein the sol is transformed into a gel upon installation in the nasal cavity is a suitable approach for a longer naso-residence time [23]. Different mechanistic approaches are available to trigger sol-to-gel phase transitions in the nasal cavity viz., ion-triggered, temperature-dependent, or pH-responsive system [24].

Therefore, the present study focuses on the development of a lipid-based self-nanoemulsifying system of naringin reconstituted in an in situ gelling biodegradable vehicle for effective nose-to-brain delivery. The formulation utilizes nasal mucosal secretions to undergo ion-triggered gelation to form a cross-linked gellan matrix, that further enables prolonged residence time at the mucosal site due to mucoadhesion and decreased mucociliary clearance. The developed system was evaluated for several physicochemical, mechanical and rheological characteristics making it optimum for nasal delivery. Further, the capability of ex vivo diffusion through sheep nasal mucosa, in vitro biocompatibility studies along with evaluation of brain targeting efficiency strengthened the safety and applicability of the platform technology for small lipophilic phytochemical.

## 2. Materials and Methods

Naringin was purchased from Sigma-Aldrich (Bangalore, India). Acrysol K-140, Acrysol K-150 (Propylene glycol-Polyoxyl 40-hydrogenated castor oil) and Kelcogel CG-HA were gifted by Coral Pharma Chem. (Ahmedabad, India) and Signet Chem Corporation (Mumbai, India). Capmul MCM (Glyceryl caprylate/caprate) and Captex 355 were gifts from Abitech Corporation, Columbus, OH, USA. Capryol 90 (Proyle glycol monocarylate), Labrafac CC (Caprylic caric triglyceride) and Labrasol (Caprylocaproyl macrogol-8 glycerides) were gifted by Gattefosse India Pvt. Ltd. (Mumbai, India). Tween 20 and Tween 80 were purchased from Molychem (Mumbai, India). Cremophor EL and Cremophor RH 40 were received as gift sample from BASF (Mumbai, India). Polyethylene glycol 400 and propylene glycol were purchased from LobaChemie Ltd. (Mumbai, India). Ethanol, sodium chloride, potassium chloride and calcium chloride were purchased from Research Lab Fine Chem Industries (Mumbai, India). All these chemicals were of analytical grade and used without any further purification. 3-(4,5-dimethylthiazol-2-yl)-2,5-diphenyl tetrazolium bromide (MTT), Dulbecco’s Modified Eagle’s medium (DMEM), Eagle’s Minimum Essential Medium (EMEM), Fetal bovine serum, Antibiotic solution (100×), Trypsin-EDTA Solution and Hank’s Balanced Salt Solution (HBSS) were purchased from Himedia, Mumbai, India.

### 2.1. Formulation of Naringin Self-Emulsifying System

The solubility of naringin was determined in various oils/modified oils (Acrysol K150, Acrysol K140, Captex 335, Olive oil, Hariol 538, Capmul MCM, Capryol 90 and Labrafac CC), surfactants (Cremophor EL, Cremophor RH 40, Labrasol, Tween 20 and Tween 80) and co-solvents (ethanol, polyethylene glycol 400, propylene glycol and Transcutol HP) using shake flask method [25]. An excess of naringin was added to 1 mL of the above-mentioned vehicles and the resulting suspensions were shaken on a flask shaker (Kytose EOS-10M, Electrolab, Mumbai, India) at room temperature for 2 days and centrifuged at 3000 rpm for 15 min (Spinwin MC 01, Tarson, Mumbai, India). The clear supernatant was analyzed for the content of naringin using the validated RP-HPLC method. Determinations were carried out in triplicate.

Based on the solubility data, a series of SEDDS pre-concentrate were prepared comprising Acrysol K140 as oil phase, Tween 80 as surfactant and Transcutol HP as co-solvent. The concentrations of oil: (surfactant and cosolvent; Smix) were varied from 1:1 to 9:1 whereas, surfactant: cosolvent proportions were varied as 1:1, 1:2 and 2:1. Naringin 100 mg/mL was added to all preconcentrates and mixed thoroughly for 30 min. Homogeneous mixtures of surfactant, oil and co-solvent at a given volume ratio were mixed in a glass vial on an orbital shaker for 10 min.

### 2.2. Construction of Pseudo-Ternary Phase Diagrams

The mixtures of oil and surfactant/co-surfactant at certain volume ratios were diluted with water in a drop-wise manner. For each phase diagram at a specific ratio of surfactant/co-surfactant 1:1, 2:1 and 1:2 (*v*/*v*), a transparent and homogeneous mixture of oil and Smix was formed by the shake flask method. Nine different combinations of oil and Smix, 1:9, 2:8, 3:7, 4:6, 5:5, 6:4, 7:3, 8:2 and 9:1, were made so that maximum ratios were covered for the study. Each mixture was titrated with double distilled water. During titration, samples were stirred to ensure homogeneity and visually monitored for phase clarity. Pseudo-ternary phase diagrams were plotted considering clear and isotropic samples falling in thenanoemulsion region. [26].

### 2.3. Characterization of Naringin Loaded Self-Emulsifying Systems

#### 2.3.1. Droplet Size Analysis, Polydispersity Index, Zeta Potential, pH and Electron Microscopy

The droplet size and polydispersity index (PDI) of naringin self-emulsifying systems were determined by photon cross-correlation spectroscopy. Formulations were diluted with double distilled water to ensure that the light scattering intensity was within the instrument’s sensitivity range. Each sample was analyzed for dynamic light scattering at room temperature. Mean droplet size and the polydispersity of the of the formulations were tested on Nanophox size analyzer (Sympatec, Clausthal-Zellerfeld, Germany). The droplet size distribution was expressed in terms of the polydispersity index. Droplet size and PDI were measured at regular intervals for 3 months to check stability. Zeta potential was measured on a zeta meter (Delsa Nano C, Beckman Coulter, Tokyo, Japan).

pH of the formulation was measured using pH meter (Eutech instruments, Singapore, Singapore).

For scanning electron microscopy (SEM), the diluted nanoemulsion (10 μL) was placed on carbon conductive adhesive tape mounted on the specimen stub. The mounted sample was frozen at −190 °C in liquid nitrogen and transferred to the preparation chamber, maintained at −130 °C and sublimed at −90 °C for 10 min, followed by coating with platinum. It was then transferred to the SEM chamber for viewing at −150 °C with an accelerating voltage of 5.0 kV (JSM-7600F Field Emission Gun (FEG) SEM equipped with Cryo unit (PP3000T) by Quorum Technologies, Laughton, UK). For transmission electron microscopy (TEM), the nanoemulsion was mounted on a carbon-coated formvar grid and stained with neutralized 2% phosphotungstic acid. The grid was dried and imaged on high-resolution TEM (JEM 200, JEOL).

#### 2.3.2. Thermodynamic Stability

The nanoemulsifying preconcentrates were analyzed for heating-cooling stability by storing them at 4 °C and 45 °C 48 h at each temperature for s 3 cycles. The formulation was monitored visualy for precipitation if any after centrifugation for 10 min at 3000 rpm. Similar cycles were performed at 25 °C and −25 °C for freeze-thaw stability. The emulsion samples were gradually heated and observed for any turbidity or phase separation, and the corresponding tempertaures were moted [26].

#### 2.3.3. RP-HPLC Analysis of Naringin

Naringin content was estimated using the validated RP-HPLC method (Supplementary Data File, Section S5 HPLC method validation and Table S3). All test samples were diluted suitably with mobile phase and the chromatographic separation was performed using an isocratic elution. The mobile phase consisted of a mixture of potassium phosphate buffer (10 mM, pH adjusted to 3.6 using dilute orthophosphoric acid) and acetonitrile (25:75) and delivered at a flow rate of 1 mL/min. The HPLC system consisted of a pump (Jasco PU-2080 Plus, Intelligent HPLC pump, Tokyo, Japan) connected to Detector (Jasco 2075, Intelligent UV–vis detector, Tokyo, Japan). The separation was carried out at 20 °C, on a reversed-phase C18 column (Qualisil^®^ BDS, 250 × 4.6 mm, 5 μm particle size). An injection volume of 20 μL was used. Detections were carried out at 284 nm. The method was validated for accuracy, precision, specificity and solution stability.

### 2.4. Formulation of Naringin Loaded In-Situ Gelling Nanoemulgel, Characterization for Gelling Capacity, Droplet Size, In Vitro Release Beahavior and FTIR Analysis

A series of gels with varying amounts of gellan gum (0.2–1.5% *w*/*v*) were dispersed into hot water at 75 °C and maintained further at 75 ± 2 °C for 10 min. to form a clear solution. On cooling, naringin self-emulsifying preconcentrate (10% *v*/*v*) containing naringin 100 mg/mL were added to the clear solution of gellan gum at ambient temperature. The dispersions were mixed well to obtain final nanoemulgel formulations.

To determine the gelation capacity the above dispersions were mixed with various amounts of simulated nasal fluid (SNF) (50–250 μL) in the transparent glass tubes [27]. The SNF was prepared by dissolving sodium chloride, calcium chloride and potassium chloride, 2.1925 g, 0.145 g and 0.745 g, respectively, in 250 mL of double distilled water [24]. The tubes were sealed with parafilm and heated in a constant temperature water bath. The temperature of the water bath was increased in increments of 2 °C and left to equilibrate at each new temperature. The formulations were examined for gelation which was said to have occurred when the meniscus would no longer move upon tilting through 90°. Measurements were done in triplicate [28].

All the formulations with varying concentrations of gellan gum were tested for in vitro release behavior. In situ gelling nanoemulgel (1 mL) was transferred to a Float-A-Lyzer (G2, Spectrum, Repligen, Waltham, MA, USA) and introduced into covered beakers containing 500 mL release media (SNF) and stirred at 100 rpm on a magnetic stirrer. Naringin released was analysed at predetermined time intervals by withdrawing 0.5 mL of release media. The volume of release media was maintained by replacing equal amount of release media immediately after sampling. Naringin content in release samples was determined by using the validated RP-HPLC method.

Fourier Transform infrared (FTIR) analysis was performed on a spectrophotometer (Bruker, Ettlingen, Germany) using KBr. The frequency range was from 4000 to 400 cm^−1^.

Droplet size of the optimum composition was measured after diluting the formulation suitably with water, following the method described in Section 2.3.1. The size measurement was performed periodically after storing the sample at ambient temperature to check the stability.

### 2.5. Rheological Profiling of Naringin Loaded In-Situ Gelling Nanoemulgel

The viscosity of the nanoemulsified naringin formulation was measured using a dynamic shear rheometer (Physica MCR 301, Anton Paar, Ashland, VA, USA), using the cone and plate assembly. Samples before mixing with SNF (pre-gelation) were analyzed at ambient temperature, whereas formulation after external gelation by mixing it with SNF (post-gelation) was tested at 37 °C. The samples were subjected to shear rates ranging from 0.1 to 100 s^−1^. The amplitude sweep measurement was carried out on both samples to analyze the linear viscoelastic region. The frequency sweep measurements were carried out to understand the behavior of the material based upon storage or elastic modulus (G′, Pa) and loss or viscous modulus (G″, Pa) [29,30]. A mean of three independent measurements was recorded.

### 2.6. Texture Profile Analysis of Naringin Loaded In-Situ Gelling Nanoemulgel

The textural analyzer (TA.XT2i plus, Stable Micro Systems, Surrey, UK) equipped with a cylinder probe was inserted into the test samples (50 mL) maintained at ambient temperature and 37 °C for pre-gelation and post-gelation samples, respectively. The analytical probe was inserted at a predetermined rate of 1 mm/s to a fixed depth of 5 cm. Mechanical parameters namely adhesiveness, cohesiveness and hardness were derived from the obtained force-time curve generated by the Texture Exponent software (V5, Stable Micro Systems, Surrey, UK). A mean of three independent measurements was recorded.

### 2.7. Ex Vivo Diffusion Study

#### 2.7.1. Preparation of Sheep Nasal Mucosa and Experimental Setup

Ex vivo diffusion studies of naringin-loaded in situ gelling nanoemulgel were carried out through freshly isolated sheep nasal mucosa using a vertical jacketed Franz diffusion cell with a receptor volume capacity of 12.5 mL. Within 10 min of the killing of the sheep at the local slaughter house, the mucosa was carefully removed, immediately immersed in ice-cold phosphate buffer saline pH 6.4 for 15 min and was aerated [24]. Mucosal specimen having thickness 0.12 cm and effective surface area 3.14 cm^2^ was used for permeation study [28]. Within 30 min of removal, the excised nasal mucosa was mounted in the diffusion cell with the mucosal surface facing the donor chamber and serosal side facing the receptor chamber. The mucosal side and serosal side of mucosa were filled with the SNF and phosphate buffer saline, respectively and bubbled. After 10 min. preincubation period to reach to 34 °C, diffusion experiments were initiated with freshly degassed phosphate buffer saline prewarmed to desired temperature. Constant circulation water bath maintained the temperature of system at 34 ± 1 °C. The test sample was loaded on the mucosa after 15 min stabilization period. Uniformity in the receptor chamber was ensured through magnetic stirring of teflon coated magnetic bar.

#### 2.7.2. Intranasal Naringin Delivery

The surface of the nasal mucosa was treated with naringin-loaded in situ gelling nanoemulgel by placing the sample in the donor chamber. Aliquots from the receptor chamber (250 μL) were withdrawn every 30 min interval for 6 h. The volume of the receptor chamber was maintained by replacing with the equal volume of fresh medium maintained at the same temperature. The samples withsrawn from the receptor chamber were stored at 4°C until analyzed for naringin content. After a 6 h permeation experiment, the mucosal surface was washed and sonicated for 30 s with 1 mL SNF to recover the unabsorbed naringin from the surface. For comparison, another gel consisting of a dispersion of naringin with equivalent concentration was also prepared by thoroughly dispersing naringin in gellan gum solution. Diffusion study samples were analyzed by the validated RP-HPLC method as described above. All measurements were carried out in triplicate.

The ex vivo nasal mucosal permeation profile of naringin was fitted to the non-steady state solution to Fick’s second law (Equation (1)) for a single layer membrane to determine the permeability characteristics of naringin.

(1) Qt=Cd.(KL){DL2t−16−2π2∑n=1∞(−1)n/n2.exp(−DL2.n2π2.t)}

where *Q_t_* is the cumulative quantity of naringin absorbed through the mucosa as a function of time. *C_d_* is the initial total donor chamber concentration of naringin.

Steady state flux (*J_ss_*, µg/cm^2^·h) was calculated as the amount of naringin passing across 1 cm^2^ of the permeation membrane per unit time (Equation (2)).

(2) $$J_{ss}=\Delta Q_{t}∕\left(\Delta t\times S\right)$$

where Δ*Q_t_/S* is the cumulative drug permeation per unit of mucosal surface area (µg/cm^2^), t is time expressed in h. *Jss* was calculated by plotting the cumulative amount of naringin (µg) permeated per unit area against time (h) and slope of the linear portion of the curve was considered as steady state flux.

Apparent permeability coefficient (*P_app_*, cm/h) and Steady state diffusion coefficient (*D*) were calculated according to the Equations (3) and (4).

(3) $$P_{app}=J_{ss}∕C_{d}$$

(4) D=Papp×LK

*K* is the partition coefficient of the naringin (log *P*) and *L* is the diffusion path length [28].

### 2.8. Biocompatibility and Toxicity Analysis

#### 2.8.1. In Vitro Testing for Biocompatibility with L929 Cell Line

The in situ gelling nanoemulgel of naringin was tested for biocompatibility in the L929 mouse fibroblast cell line (National Centre for Cell Sciences, NCCS, Pune, India) [31]. The cells were maintained in DMEM and distributed in 96-well plates (1 × 10^4^ cells per well) for 24 h in a 5% CO_2_ atmosphere at 37 °C. After this period, the medium was removed and the adhered cells were treated with formulations (4 dilutions taking 0.2 g/mL of formulation in DMEM as 100% and further dilution with DMEM to obtain 50%, 25% and 12.5%, *n* = 6) under the same incubation conditions. The growth of untreated cells was considered as 100% viable and was considered as control. MTT (1 mg/mL in HBSS) reagent was used for MTT assay (ISO 10993-5). After addition of MTT reagent the cells were incubated further for 3 h. The medium was removed and DMSO was added to dissolve the formazan crystals. The optical density was measured after 30 min at 570 nm on a microplate reader. The viability (%) was calculated by comparing with the absorbance of untreated cells.

#### 2.8.2. Assessment of Local Toxicity on the Nasal Mucosa

To determine the pathological changes occurring in cell morphology and tissue organization, histopathology was performed on sheep nasal mucosa treated with naringin loaded in situ gelling formulation for 6 h. For comparison, normal mucosa treated with phosphate buffer saline (pH 6.8) was considered as negative control and mucosa treated with 100 μL 37% *v*/*v* nitric acid for 2 h was considered as a positive control. Nasal mucosa specimens were stored in 10% formalin in phosphate-buffered saline, embedded in paraffin and stained with hematoxylin and eosin. Sections were observed under a microscope and analyzed by a pathologist blinded to the experimental conditions.

### 2.9. Pharmacokinetics and Brain Targeting Efficiency of Naringin Loaded In-Situ Gelling Nanoemulgel

In vivo studies were performed in Wistar rats of either sex. The experimental protocol was approved by the Institutional Animals Ethics Committee (IAEC) of Vidya Siri College of Pharmacy, Bangalore, India (Approval number- VSCP/EC/2208/2020/2 and approval date 22 August 2020) and the experiment was carried out according to the guidelines of Committee for the Purpose of Control and Supervision on Experiments on Animals (CPCSEA) for experimental animal care (Approval no. VSCP/EC/2208/2020/2). Rats weighing 200–220 g were housed in standard wire mesh plastic cages in a room maintained at 22 ± 0.5 °C and day/night cycle of 12 h each. Animals were given standard pellet food and water ad libitum. Rats were divided into different 3 groups, i.e., Groups A, B and C (*n* = 6, at each time point). Group A was administered with naringin suspension via intranasal route (10 μL, 0.5 mg/kg) and Groups B and C were administered with naringin in situ gelling nanoemulgel via intranasal route and intravenous route (10 μL, 0.5 mg/kg), respectively. Blood samples (0.2 mL) were collected by cardiac puncture and rats were sacrificed at 0.5, 1, 1.5, 2, 4 and 6 h time intervals. The blood samples were centrifuged in the microcentrifuge tubes containing EDTA. for 10 min at 5000 rpm and 4 °C. The plasma was stored in deep freezer (−80 ± 5 °C) for further HPLC estimation. Brains were collected and rinsed with normal saline to make it free from adhering fluid and homogenized. Brain homogenates were centrifuged and concentration of the naringin in brain and plasma was analyzed. Various parameters like AUC0-6, brain targeting efficiency and direct transport percentage were further calculated. The drug targeting efficiency was estimated using following Equation (5).

(5) $$Drug\;targeting\;efficiency=\frac{\left[\left(AUC_{Brain}\right)/\left(AUC_{Blood}\right)\right]intranasal\;}{\left[\left(AUC_{Brain}\right)/\left(AUC_{Blood}\right)\right]intrvenous}\times 100$$

Direct transport percentage was calculated using following Equation (6).

(6) $$Direct\;transport\;percentage=\frac{\left(B_{in}\right)-\left(B_{x}\right)\;}{\left(B_{in}\right)}\times 100$$

where, $B_{x}$ = ($B_{iv}$/$P_{iv}$) × $P_{in}$.

$B_{x}$ is the brain AUC fraction contributed by systemic circulation through blood brain barrier following intranasal administration. $B_{iv}$ is the brain AUC following intravenous administration. $P_{iv}$ is the blood AUC following intravenous administration. $B_{in}\;$is the brain AUC following intranasal administration. $P_{in}\;$is the blood AUC following intranasal administration.

Nasal bioavailability was calculated using following Equation (7).

(7) $$Nasal\;bioavailability=\left(AUC_{intranasal}\right)/\left(AUC_{intravenous}\right)\times 100$$

## 3. Results and Discussion

Intranasal drug delivery, as a promising non-invasive alternative route of administration for treating CNS disorders, offers several clinical benefits such as ease of accessibility and administration that eventually leads to improved patient compliance for chronic therapies. The positive outcome of brain targeting through intranasal delivery of phytoconstituents has been validated earlier [27]. However, simple solutions for intranasal instillation suffer the drawback of poor bioavailability due to extremely low aqueous solubility of phytoconstituents and short residence at the site of absorption due to rapid mucociliary clearance We addressed both these drawbacks by developing a self-emulsifying nanosystem with high loading of hydrophobic constituent and an in situ gelling hydrogel vehicle to prolong the residence time at nasal mucosa.

### 3.1. Designing of Naringin Self-Emulsifying Nanosystem

The selection of excipients was done based on solubility and miscibility studies. If drug solubility is adequate there are fewer chances of precipitation upon dilution with aqueous media. Co-solvents such as PEG, propylene glycol and alcohols help to solubilize large quantities of a drug in the oil phase. The high drug solubility in the oil phase is an important parameter for the successful delivery of drugs through self-emulsifying delivery systems. Therefore, the equilibrium solubility of naringin was estimated in different lipid excipients.

**Figure 1:**
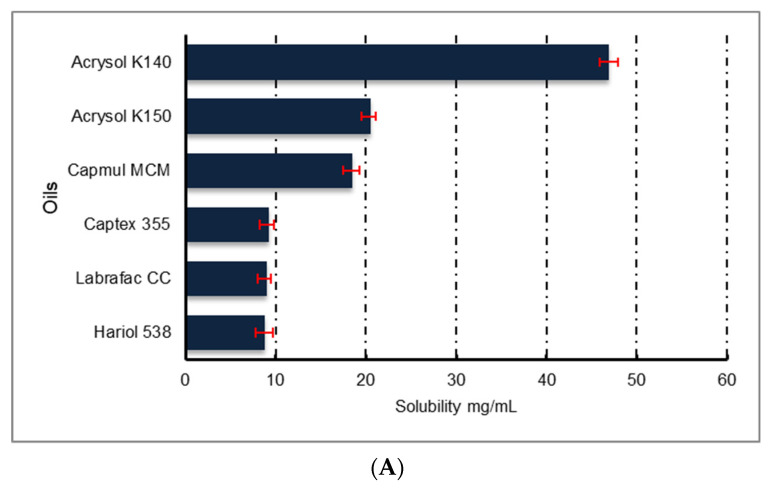

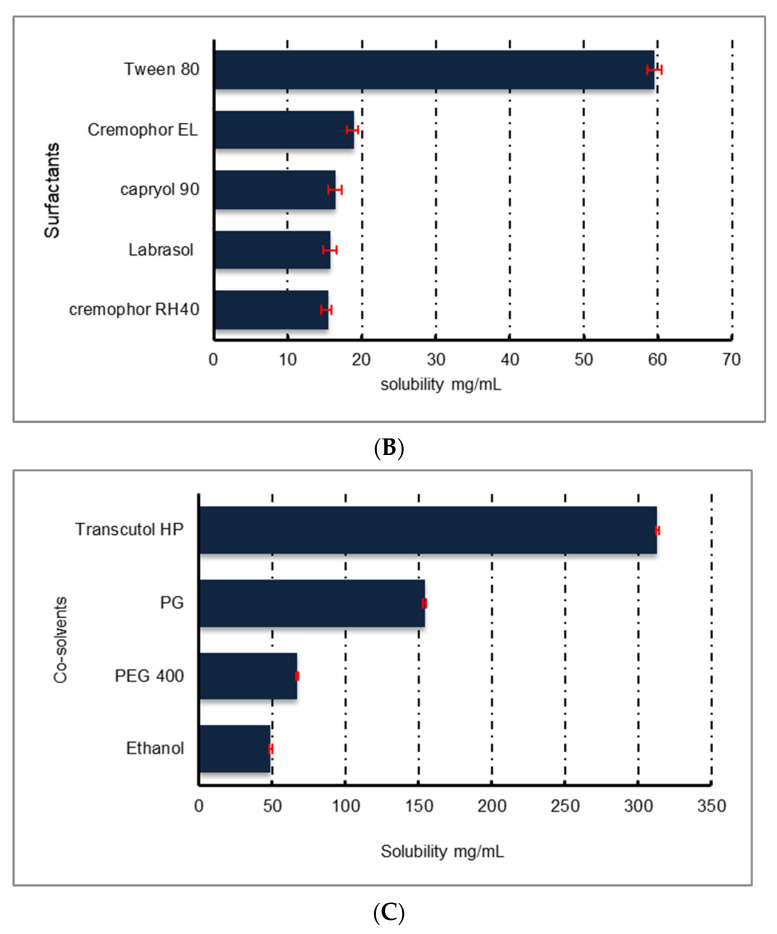
Solubility of naringin in various (**A**) oils, (**B**) surfactants and (**C**) cosolvents, (*n* = 3 ± SD).

Naringin showed the highest solubility in Acrysol K140, Tween 80 and Transcutol HP (Figure 1) among the several oils, surfactants and co-solvents evaluated, respectively. Hence, these three ingredients were selected for further optimization.

### 3.2. Preparation of Pseudo-Ternary Phase Diagrams

**Figure 2:**
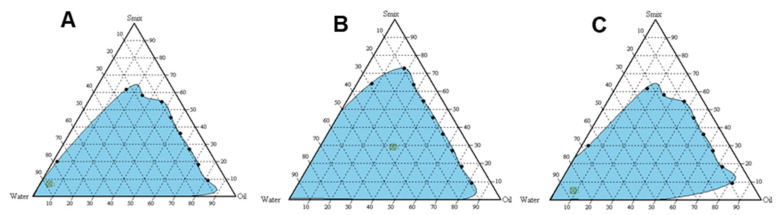
Pseudo-ternary phase diagrams indicating the efficient self-emulsification region. (**A**) Smix 1:1, (**B**) Smix 1:2; (**C**) Smix 2:1.

The higher emulsifying potential is another important criterion to look after when dealing with self-emulsifying systems. The branched structure of surfactant promotes greater penetration of oil and thus enables better emulsification capacity. Thus, the preconcentrates combination having the better emulsifying ability at a lower proportion of surfactant and higher drug loading potential is the key to successful self-emulsifying formulation development [32]. In addition, uniform droplet size distribution allows better emulsification capacity of the preconcentrate combinations. Among three surfactant and cosolvent combinations evaluated, emulsion formation was found to be greater with Smix = 1:2, the system could solubilize much of the oily phase. Thus, based on the optimization parameters, Smix at 1:2 was selected for loading of naringin into the preconcentrates (Figure 2).

### 3.3. Physicochemical Characterization of Nanoemulsified Formulations

#### 3.3.1. Droplet Size, Polydispersity Index, Zeta Potential, pH and Electron Microscopy

**Figure 3:**
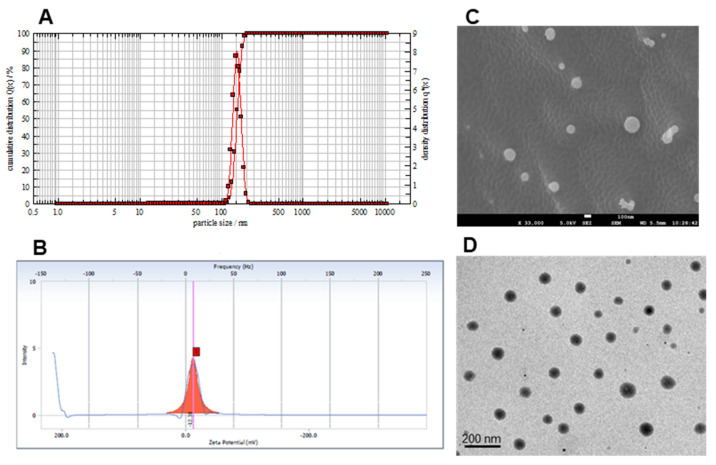
Droplet size analysis (**A**), Zeta potential (**B**), SEM image (Scale 5mm = 100 nm) (**C**) and TEM image (Scale 1 cm = 200 nm) (**D**) of nanoemulsion of naringin obtained after dilution of self-emulsifying preconcentrate of naringin with water.

The globule size was found to less than 200 nm for all the formulations prepared with Acrysol K140 ranging from 10–90 (*v*/*v* %). Droplet size was found to increase with an increase in oil content in the formulation. Mean droplet size of naringin loaded formulation with Acrysol K140:Smix 9:1 was selected for drug loading based on the particle size. The particle size of the self-emulsifying concentrate of naringin was found to be 152.03 ± 4.6 nm with a polydispersity index of 0.23 when diluted with water (Figure 3A).

This indicates uniform and narrow droplet size distribution of the prepared self-emulsifying nanosystems, which was also evident from the SEM and TEM images (Figure 3C,D). In addition, it has been observed that the small droplet size of the dispersed oil phase implies faster drug release from the formulation due to increased surface area [33] and also enhances emulsification capacities [34]. The product was stable over a period of 3 months. There was no significant difference in size and PDI (Supplementary Data File; Results: S1, Figure S1). The surface charge of the emulsion formed from self-emulsifying systems is believed to play an essential role in its stability [35]. Using Acrysol K140 as the oil and Tween 80 as the surfactant with co-solvent Transcutol HP, the emulsion obtained was negatively charged with a zeta potential of −15 mV (Figure 3B). The anionic groups of the fatty acids and glycols present in the oil, surfactant and co-surfactant impart a negative charge on the self-emulsifying formulations [36]. For small molecules, the stability is considered to be high if the zeta potential value is high, accordingly the droplets will oppose the aggregation.

The nanoemulsion showed pH 6.5 ± 0.2 (*n* = 3 ± SD) and after converting it into nanoemulgel, the pH was found to be 6.7± 0.3(*n* = 3 ± SD). This pH is in the range of pH of nasal mucosa making it suitable for nasal application.

#### 3.3.2. Thermodynamic Stability of Nanoemulsified Formulations

The cloud point is a critical feature of emulsion systems that signifies thermodynamic stability and needs to be studied to avoid the selection of metastable emulsion. Metastable emulsion might result in instability and may become therapeutically less effective upon contact with a biological fluid. The thermodynamic stability of self-emulsifying nanosystem is achieved by tuning the interfacial tension to comparable or even lower than the entropy of dispersion which results in the free energy of the system to become zero or negative [37].

When the temperature is higher than the cloud point, irreversible phase separation occurs and the cloudiness of the preparation would have a negative impact on drug absorption, because of the dehydration of the components. Thus, the cloud point for self-emulsifying systems must be above 37 °C in order to avoid phase separation upon reaching the gastrointestinal tract of the human body [32]. The cloud point of the developed self-emulsifying nanosystem was found to be greater than 70 °C, suggesting its stability of formulation at the body temperature as well as accelerating stability test conditions. Naringin loaded nanoemulsified formulation showed no precipitation and phase separation indicating its stability.

### 3.4. Formulation of Naringin Loaded In-Situ Gelling Nanoemulgel and Characterization for Gelling Capacity, Droplet Size, In Vitro Release Behavior and FTIR Analysis

Ideally, the intranasal in situ gelling hydrogel should stabilize the nanoemulsion in the liquid state and additionally should extend the duration of action by gelling in presence of nasal fluid at the site of absorption. Therefore, we selected gellan gum as it is heat-stable, has a high melting, biodegradable and exhibits strong gelling ability. Gels produced by gellan polysaccharide are highly clear and biocompatible making it an ideal hydrogel for intranasal instillations.

Mono- as well as di-valent cations, trigger gelation of anionic gellan gum by binding to coordination sites. This causes a reduction in electrostatic repulsions in the helices of gellan gum resulting in a structurally cross-linked three-dimensional network. Cations present in biological fluids, primarily sodium, potassium along with traces of calcium trigger the formation of cross-linked gellan matrix over the nasal mucosa. Gelling ability of the naringin nanoemulsion loaded gellan hydrogel at varying concentrations (0.2–1.5% *w*/*v*) was tested by mixing with SNF. It is a very critical parameter to study because, in the presence of self-emulsifying components and naringin, gellan hydrogel may show a lower gelling tendency. The minimum concentration of gellan in naringin nanoemulsion loaded formulation that could produce gelation of suitable viscosity was found to be 1.1% *w*/*v*. Above this concentration, the gel was found to be too viscous to handle and process. (Data is shown in Supplementary Data File, Results S2, Table S1).

In vitro release behavior was tested for various naringin nanoemulsion loaded gellan hydrogel with varying concentrations of gellan ranging from 0.2 to 1.5% *w*/*v*. The results of the same are added in Supplementary Data File (Results S3, Figure S2. And Table S2).

Droplet size analysis of the optimum composition over a period of time and the FTIR data are shown in Supplementary Data File (Results S1, Figure S1B and Results S4, Figure S3, respectively).

### 3.5. Rheological Profiling of Naringin Loaded In-Situ Gelling Nanoemulgel (Pre and Post Gelation)

**Figure 4:**
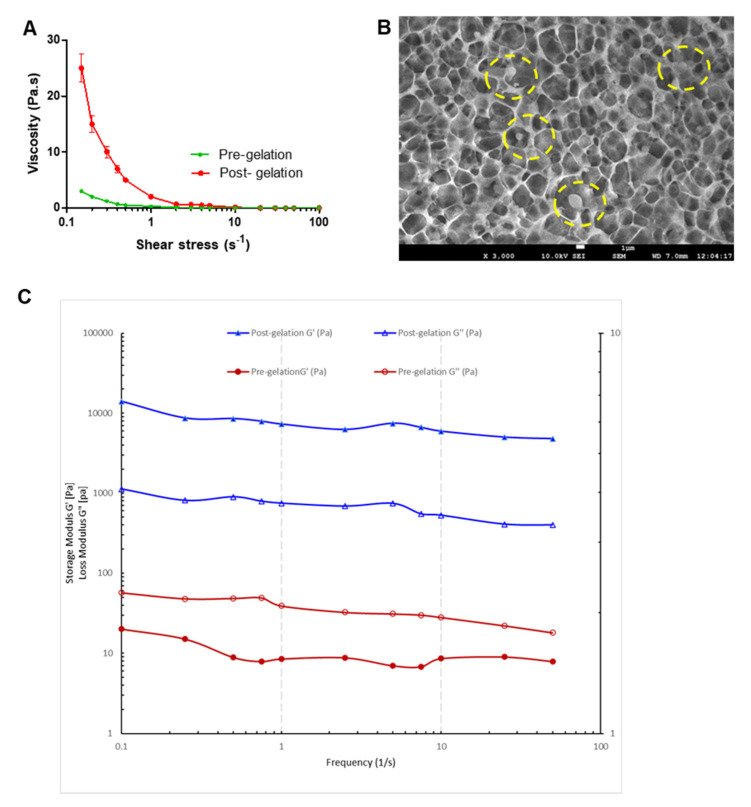
(**A**) Rheological profiling of naringin loaded in situ gelling nanoemulgel depicting change in viscosity with increase in rate of shear before mixing with SNF (Pre-gelation) and after mixing with SNF (Post-gelation). (**B**) SEM image of naringin loaded in situ gelling nanoemulgel after mixing with SNF showing three-dimensional network entrapping nanoemulsion droplets (5 mm = 100 nm). (**C**) Frequency sweep evaluation showing comparative G′ (storage modulus, solid markers) and G″ (loss modulus, hollow markers) of the naringin loaded in situ gelling nanoemulgel pre- and post-gelation.

Viscosity measurements (Figure 4A) show that the viscosity of nanoemulgel was rapidly increased (at low shear rates) after being mixed with SNF showing gelation of gellan in presence of cations.

This verifies the in situ gelation when the formulation will be instilled in the nasal cavity in vivo. SEM imaging (Figure 4B) shows a cross-linked gellan matrix with embedded nanoemulsion droplets of naringin. A decrease in viscosity with the applied shear force indicates the shear-thinning property of the hydrogel. This will ensure that the nanoemulgel will flow easily after shaking the container making it fluid enough to allow easy instillation. This is a typical non-Newtonian flow behavior of gellan hydrogel in which the complex viscosity decreases as shear force increases.

### 3.6. Rheological Profiling of Naringin Loaded In Situ Gelling Nanoemulgel System

The rheological behavior of the developed in situ gelling product affects several characteristic features viz. flow from the container during instillation in the nasal cavity, intended residence time of the product at the application site and spreadability and adhesivity of the product over the mucosal tissue. Traditional aqueous nasal solutions being less viscous and flowable liquids tend to have poor adherence in the nasal mucosa wherein the olfactory and trigeminal nerves are located. Favorable rheology can ensure that in situ intranasal formulations can be easily administered and solidify in presence of cations from the nasal fluid to form a stable hydrogel. This change in the physical structure of the formulation prolongs the retention time and helps enhance bioavailability. Therefore, it is crucial to determine the rheological behavior of the developed product.

Moreover, measurement of only viscosity of the gel as the rheological parameter [38,39] does not yield relevant data for the gel. Disruption of the gel structure upon high shear insists upon the need for a relevant analytical tool for studying gel rheology. Thus, a more appropriate approach is to carry out oscillatory measurements wherein the oscillating angle is kept low, to ensure no alteration in the gel structure occurs during rheological measurements.

A frequency sweep test was carried out to evaluate the viscoelastic character of the pre- & post- gelation in situ nanoemulgel by applying sinusoidal shear stress to the in situ nanoemulgel sample. With the application of small oscillating strain to the in situ nanoemulgel samples, two important dynamic moduli viz., the storage modulus, G′, an index of the elasticity and the loss modulus, G″, reflective of the vicious attributes, were determined. Dynamic frequency analysis in the linear viscoelastic regime is shown in Figure 4C. The relationship between G′ and loss modulus (G″) reflects the change in viscosity and elasticity that results in a varied characteristic of a material [40].

Extended retention time at the site of instillation along with controlled and sustained drug release demands an elastic gel system [41]. The storage or elastic modulus, G’, measures the stored energy, demonstrating the elastic behavior of the solid-like material [42]. As depicted in Figure 4C, the post-gelation in situ nanoemulgel exhibit a higher value of the storage modulus G′ as compared to that of the loss modulus G″ (G′ > G″). This indicates a predominantly elastic behavior of the in situ nanoemulgel, post-gelation. Meanwhile, pre-gelation in situ nanoemulgel formulation exhibit G″ > G′, i.e., loss modulus higher than storage modulus. This is because the loss modulus G″ signifies the viscous portion of the viscoelastic material or the liquid-state behavior of the sample. Internal friction between the molecules and particles in a fluid contributes to the viscous behavior of the liquid state. Thus, in a liquid state, the stored energy is used by the internal friction processes and is dissipated as heat. Contrary to this, the elastic portion of the deformed material, in an elastic material, stores the energy. Storage modulus or G′ embodies this stored deformation energy while the loss modulus G″ exemplifies the deformation energy which is dissipated or lost due to internal friction in a flowing fluid [41,43]. Thus, in the present study, post-gelation in situ nanoemulgel is a viscoelastic solid that has G′ > G″ (higher storage modulus than loss modulus) (Figure 4C). While the pre-gelation in situ nanoemulgel represents a viscoelastic liquid that has G″ > G′ (higher loss modulus than storage modulus) due to the absence of strong bonds between the individual molecules.

Furthermore, upon the frequency sweep enhancement, the storage modulus G′ of the in situ nanoemulgel does not significantly change, indicating the in situ nanoemulgel stability [44,45].

### 3.7. Texture Profile Analysis of Naringin In Situ Gelling Hydrogel

**Figure 5:**
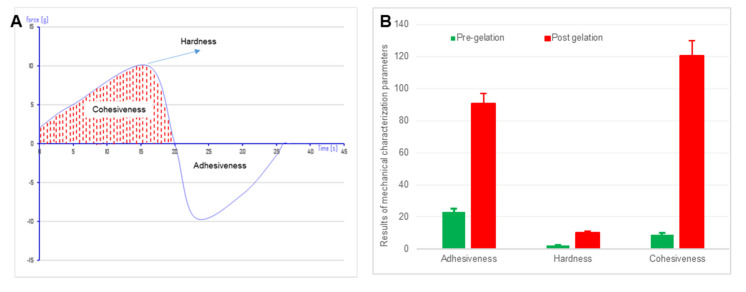
(**A**). Representative graph of force versus time relationship of naringin loaded in situ nanoemulgel post-gelation showing hardness (g), cohesiveness (g.sec, Area under curve; Dotted) and adhesiveness (g.sec, Area under curve; Plain) and (**B**). Bar graph representing comparison of various mechanical characteristics of naringin loaded in situ nanoemulgel, pre and post gelation namely adhesiveness (g.sec), hardness (g) and cohesiveness (g.sec). Data represents mean of 3 independent determination. Error bars indicate SD.

In designing an optimal intranasal formulation, particularly in respect to prolonged retention time at the site of administration for hydrogels destined for the treatment of CNS disorders, a balance between adhesiveness and cohesiveness of the gel needs to be assured. Texture profile analysis allows easy assessment of the mechanical properties of hydrogels from which the physics of gel structure can be interpreted. Various gel characteristic parameters like hardness, cohesiveness and adhesiveness were estimated from a force-time graph plotted using texture analysis (Figure 5A).

The maximum compressing force required for the penetration of the probe into the gel is the hardness of the hydrogel whereas, cohesiveness is the work done in the down movement of the analytical probe to deform the hydrogel. Adhesiveness of the gel is work done in detachment of the probe from the gel while moving in the upward direction represented by the valley in the force-time graph [46]. A significant increase in hardness and cohesiveness of nanoemulgel after mixing with SNF indicates in situ gelation (Figure 5B). An increase in adhesiveness will results in increased residence time of hydrogel at the site of absorption.

### 3.8. Ex Vivo Diffusion Study

**Figure 6:**
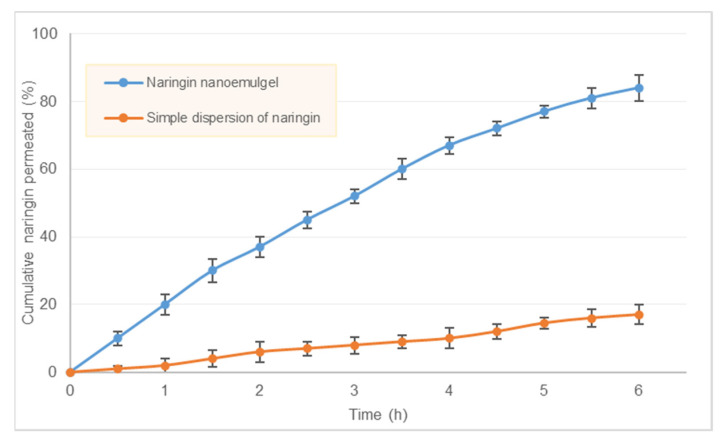
Ex vivo permeation profile of in situ gelling nanoemulgel and the simple formulation made by dispersing naringin in same vehicle. Data represent mean of 6 determinations and error bars indicate SD.

Sheep nasal mucosa offers a suitable alternative to the human nasal mucosa, as the sinus anatomy including the placement of nasal cavity, turbinates, frontal and maxillary sinuses in sheep is similar to humans. Even the histology of the sheep’s nasal mucosa is identical to that of humans. We analyzed ex vivo permeation of naringin through sheep nasal mucosa for in situ gelling nanoemulgel as well as for simple formulation in which an equivalent amount of naringin was dispersed in the vehicle. Comparative permeation of naringin from in situ gel and simple solution clearly showed enhanced permeation when formulated as nanoemulsion (Figure 6).

Approximately 84 ± 3.8% of naringin got permeated from the nanoemulgel, whereas only 17 ± 2.9% permeation was observed through simple dispersion formula. This is attributed to the poor solubility of naringin in water. For a successful delivery through the nasal route, the drug candidate needs to have adequate solubility as well as permeability. Our results indicate significant improvement in permeability of naringin achieved through solubilization in lipid-based excipients and nanosizing through the self-emulsification process. After 6 h of diffusion experiment, the amount recovered from the donor chamber accounted for 9.8 ± 2.4% and 75.5 ± 2.1% for nanoemulgel and simple naringin formulation respectively. We calculated steady-state flux (*J_ss_*), apparent permeability coefficient (*P_app_*) and the steady-state diffusion coefficient (*D*) of naringin through the sheep nasal mucosa using linear regression analysis of diffusion data. These parameters derived are tabulated in Table 1.

Small molecules cross the nasal epithelial membrane either by transcellular transport exploiting simple concentration gradients, receptor-mediated transport, or vesicular transport mechanism or by a paracellular route through the tight junction between the cells. In the case of nanoemulsion delivery, the presence of surfactants tends to fluidize emulsion droplet thus enhancing drug absorption [47]. The use of cosolvent further reduces the interfacial tension and increases the fluidity of the emulsion droplets by decreasing the energy required for deformation. The use of surfactants also causes changes in the normal lipid packing of the membrane barrier, thereby fluidizing the membrane as well. Since the components of the formulation can affect the biological tissue, it is essential to analyze the safety of the product.

### 3.9. Biocompatibility and Toxicity Analysis

**Figure 7:**
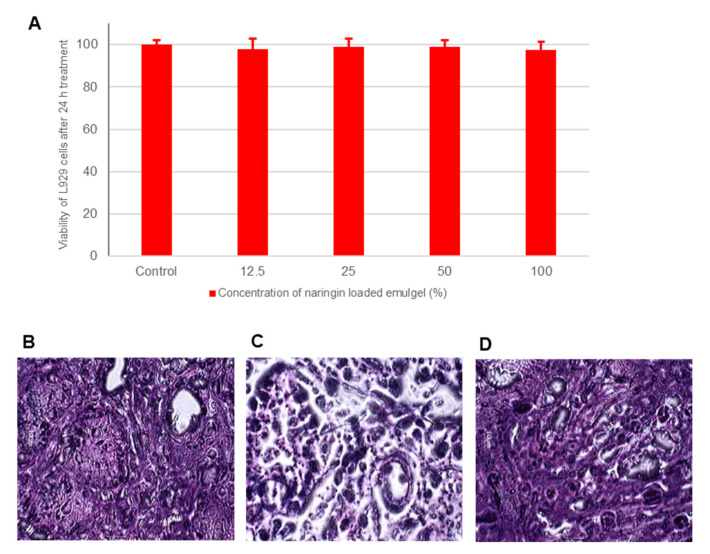
(**A**) Graph showing percentage viability versus concentration of nanoemulsion analyzed using MTT assay. Values are mean of six determinations. Error bars represent SD. Histopathological sections of nasal mucosa (100×) (**B**) Negative control: untreated mucosa, (**C**) Positive control: mucosa treated with nitric acid (37% *v*/*v*) for 2 h and (**D**) Test specimen: mucosa treated with naringin in situ gelling nanoemulgel formulation for 6 h.

In vitro test of cell toxicity provides a rough assessment of the ability of cells relevant to a determined application to survive in the presence of specific materials. The L929 fibroblast cells were treated with varying concentrations of the formulation diluted in a culture medium [48]. All four concentrations tested showed more than 98% viability for all the concentrations tested (Figure 7A).

For many wells, the viability was more than 100%, which is attributed to the presence of lipids that are used by the cells as nutrients. Overall data indicates the excellent biocompatibility of the developed product. Considering the applicability of intranasal formulations for chronic therapy, safety is of prime importance. The tissue damage of naringin loaded in situ gelling nanoemulgel was evaluated by histopathological analysis of tissue sections stained with hematoxylin and eosin. The tissue architecture and integrity of the nasal mucosa were analyzed microscopically for any inflammatory changes or toxicity. In normal nasal mucosa (untreated, negative control, Figure 7B), the structure of the mucosa was well preserved with normal characteristics of the surface pseudo epithelium. Positive control mucosa treated with nitric acid (positive control, Figure 7C) showed marked alterations in the surface pseudo epithelium. Loose, detached and vacuolated structures are visible. Nasal mucosa treated with developed nanoemulgel of naringin showed no marked alteration in the histological structure compared to the negative control (Figure 7D). There was also no evidence of necrosis or ulceration. Both the studies indicate the safety and tolerability of gellan gum-based nanoemulgel of naringin.

### 3.10. Pharmacokinetics and Brain Targeting Efficiency of Naringin Loaded In-Situ Gelling Nanoemulgel

Various pharmacokinetic parameters observed in blood and brain after administration of different dosage forms to rats as well as DTE (%) and DTP (%) are tabulated in Table 2.

High values of DTE (%) and DTP (%) for group B formulation prove the ability of the in situ gelling nanoemulgel formula to transport naringin directly to the brain with higher concentration and more rapid onset of action. Significantly higher transport of the naringin to the brain from in situ gelling nanoemulgel administered intranasally (group B, Table 2) can be attributed to its small droplet size, significantly improved solubility and rate of transport which allows transcellular transport via the various endocytic pathways of sustentacular (supporting) or neuronal cells in the olfactory membrane [49].

Gellan has been recommended as the safe biopolymer for nasal administration. Gellan gum is an anionic deacetylated exo-cellular polysaccharide that undergoes in situ gelling in the presence of cations like Ca^2+^ present in biological fluids and thus forms a three-dimensional network by complexation and hydrogen bonding with water [50]. Thus, addition of gellan gum enhance the contact between the formulation and the nasal mucosa resulting in longer nasal clearance time and prolonged residence time [24,51,52].

## 4. Conclusions

Several neurological diseases require therapies in which the drug must reach the brain overcoming the difficulties offered by the BBB. However, many drugs cannot be delivered due to the highly restrictive nature of BBB. Nevertheless, a non-invasive route along with nanotechnology remains of paramount significance for CNS delivery. The wide use of different nanocarriers has led to a wide array of techniques for improving brain targeting. In addition, naturally-derived medicinal components have the advantages of abundant resources, less side effects and improved efficacy, which can be utilized as potential drugs for the treatment of CNS diseases. The present study successfully demonstrated a naringin self-emulsifying nanosystem for augmented delivery to the brain through an intranasal route using a smart, biopolymeric, ion-triggered hydrogel as the mucoadhesive vehicle. The prepared nanoemulsion exhibited a small droplet size, sustained delivery of the naringin and enhanced diffusivity. The naringin loaded in situ gelling nanoemulgel exhibited an in situ sol-gel transition capability along with shear-thinning rheological competence. Thus, overcoming the hindrance offered by the rapid naso-mucociliary clearance. The ex vivo permeation profile suggested the improved permeation and delivery of naringin from in situ gelling nanoemulgel across the nasal mucosa after nasal administration. Further, the naringin-loaded in situ gelling nanoemulgel was found to be biocompatible and nontoxic to the nasal mucosa. Intranasal nanoemulsion is undoubtedly a promising approach for brain targeted delivery of therapeutics. The major associated challenge of nasal mucociliary clearance has already been addressed by imparting mucoadhesive characteristics. The developed phytochemical-based nanoemulgel technology provides a potential platform that could off-set the major short-comings of the conventional liquid formulations for nasal administration by virtue of its nano-sized lipid-based particles and ion-triggered gelation with natural biocompatible hydrogel polymer for the treatment of chronic CNS disorders.

## Figures and Tables

**Table 1:** Ex vivo permeation characteristics of naringin through sheep nasal mucosa for in situ gelling nanoemulgel and simple formulation (*n* = 6 ± SD).

Parameter	In Situ Gelling Nanoemulgel	Simple Formulation
Steady state flux (*J_ss_*) (μg/cm^2^·h^−1^)	45.11 *	9.02
Apparent permeability coefficient (*P_app_*) cm·h^−1^	4.5 × 10^−2^ *	0.9 × 10^−2^
Steady state diffusion coefficient (D) cm^2^·h^−1^	20 × 10^−2^ *	4 × 10^−2^

Each value represents the mean of 6 determinations. * *p* < 0.001, when compared with simple naringin formulation gel by Student’s *t*-test.

**Table 2:** Pharmacokinetic parameters, drug targeting efficiency (DTE, %), drug transport percentage (DTP, %) and nasal bioavailability of naringin formulations namely naringin suspension (group A) administered intranasally and naringin loaded in situ gelling nanoemulgel given by intranasal (group B) and intravenous routes (group C).

Group	C_max_ (ng/g)		T_max_ (min)		AUC_0–6_ (ng/g·h)		DTE (%)	DTP (%)	Nasal Bioavailability
	Blood	Brain	Blood	Brain	Blood	Brain			
A	219 ± 35	152 ± 36 ^#^	60	30	450 ± 51 ^#^	267 ± 42 ^#^	210.02	52.4	43.3
B	527 ± 31 *	450 ± 42 *^,#^	60	60	1183 ± 127 *^,#^	1892 ± 169 *^,#^	566.11 *	82.3 *	306.6 *
C	--	206 ± 23	--	60	2184 ± 289	617 ± 75	--	--	--

Data of C_max_ and AUC_0–6_ are expressed as mean ± SD, *n* = 6. * Significant difference (*p* < 0.05) when compared with group A. ^#^ Significant difference (*p* < 0.05) when compared with group C.

## Data Availability

The authors confirm that the data supporting the findings of this study are available within the article and also as Supplementary Materials.

## Supplementary Materials

The following are available online at https://www.mdpi.com/article/10.3390/pharmaceutics13081216/s1, Figure S1: Droplet size analysis and PDI at 1, 2 and 3 months, of (A) nanoemulsion of naringin obtained after dilution of self-emulsifying preconcentrate of naringin with water and (B) naringin loaded in situ gelling nanoemulgel, Figure S2: In vitro release behavior of various naringin nanoemulsion loaded gellan hydrogels with varying concentrations of gellan ranging from 0.2 to 1.5% *w/v*. Each data point is mean of 6 determinations and error bars indicate standard deviation, Figure S3: FT-IR spectra of A: Naringin, B: Gellan gum C: Gellan gum after gelation D: Naringin loaded in situ gelling nanoemulgel, Table S1: Gelling capacity of various formulations obtained after mixing of naringin loaded in situ gelling nanoemulgel containing various concentration of gellan gum with SNF, Table S2: *f*_2_ similarity factor values after comparing the release profiles of various naringin nanoemulsion loaded gellan hydrogels with varying concentrations of gellan ranging from 0.2 to 1.5% *w/v*, Table S3: RP-HPLC method validation parameters for the estimation of naringin.
