# Supplementary material for: Ion-Triggered In Situ Gelling Nanoemulgel as a Platform for Nose-to-Brain Delivery of Small Lipophilic Molecules
Source: Pharmaceutics. 2021 Aug 6;13(8):1216. doi: 10.3390/pharmaceutics13081216 (PMC8400950; doi:10.3390/pharmaceutics13081216)
Supplement: Supplementary file 1 [file pharmaceutics-13-01216-s001.zip › pharmaceutics-1306271-supplementary.pdf]

pharmaceutics

MDPI

Supplementary Materials: Ion-Triggered In Situ Gelling Nanoemulgel as a Platform for Nose-To-Brain Delivery of Small Lipophilic Molecules

Sreeharsha Nagaraja, Girish Meravanige Basavarajappa, Ranjith Kumar Karnati, Esam Mohamed Bakir and Swati Pund

S1. Droplet size and PDI measurement over a period of time

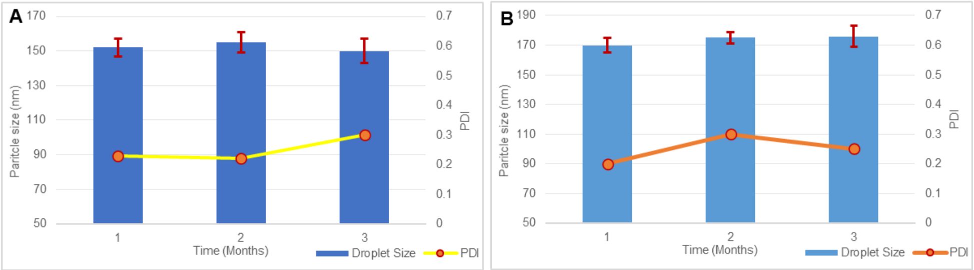

Figure S1. Droplet size analysis and PDI at 1, 2 and 3 months, of (**A**) nanoemulsion of naringin obtained after dilution of self-emulsifying preconcentrate of naringin with water and (**B**) naringin loaded in situ gelling nanoemulgel.

No significant change was observed in globule size of nanoemulsion over a period of time indicating stability of the product at ambient temperature (Figure S1A and B). Size of the droplets after gelation was more than that of emulsion. This can be attributed to the additional stabilisation of droplets due to adsorbed polymer (Figure S1B).

S2. In vitro gelation behavior

Table S1. Gelling capacity of various formulations obtained after mixing of naringin loaded in situ gelling nanoemulgel containing various concentration of gellan gum with SNF.

Gellan Concentration (%)	Temperature (°C)					
	25	27	29	31	33	35
0.2	-	-	-	-	-	-
0.4	-	-	-	-	-	-
0.6	-	-	-	-	-	-
0.7	-	-	-	-	-	-
0.8	-	-	-	-	-	-
0.9	-	-	-	-	-	-
1.0	-	-	-	-	-	+
1.1	-	-	-	-	+	+
1.2	-	-	+	+	+	+
1.3	-	+	+	+	+	+
1.4	+	+	+	+	+	+
1.5	+	+	+	+	+	+

If the formed gels showed adhesion to the tubes instead of flowing or slipping, this status was regarded as“+” to indicate gel formation. Flowing or slipping liquids were labelled as “-”.

S3. In vitro release behavior

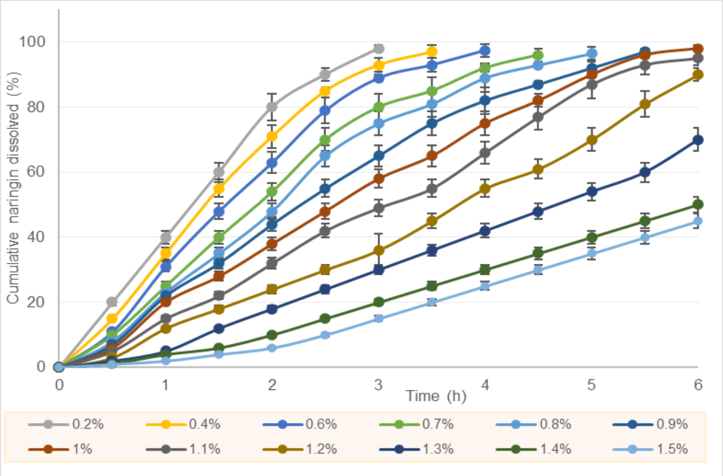

Figure S2. In vitro release behavior of various naringin nanoemulsion loaded gellan hydrogels with varying concentrations of gellan ranging from 0.2 to 1.5% *w/v*. Each data point is mean of 6 determinations and error bars indicate standard deviation.

In vitro release profiles clearly indicate the release retarding effect of increasing concentration of gellan. In situ gelling nanoemulgels with lower levels of gellan (from 0.2 to 0.6% *w/v*) showed almost complete release within 4 h.

Closeness in the release profile was analyzed using f_2 similarity factor (Table S2).

Table S2. f_2 similarity factor values after comparing the release profiles of various naringin nanoemulsion loaded gellan hydrogels with varying concentrations of gellan ranging from 0.2 to 1.5% *w/v*.

Comparison of release profiles	f_2 value
0.2% *vs* 1.1%	10.54
0.4% *vs* 1.1%	12.80
0.6% *vs* 1.1%	13.36
0.7% *vs* 1.1%	14.24
0.8% *vs* 1.1%	20.43
0.9% *vs* 1.1%	21.38
1% *vs* 1.1%	61.22
1.2% *vs* 1.1%	50.18
1.3% *vs* 1.1%	34.33
1.4% *vs* 1.1%	25.00
1.5% *vs* 1.1%	22.38

As observed from table 2, the release profiles are dissimilar when compared with that of 1.1% *w/v* gellan gum except for 1 and 1.2% *w/v* which showed f_2 similarity value 61.22 and 50.18% respectively. The release profile of the optimized batch with 1.1% *w/v* gellan gum was analyzed using various mechanistic models (zero order, first order, Higuchi, Hixon-Crowell, Korsemeyer-Peppas model, etc. The Korsemeyer-Peppas model provided the best fit ($R^2 = 0.99$) for the in situ gelling naringin nanoemulgel. The equation applied to the data was $M_t/M_\infty = Kt^n$, where, K is kinetic constant incorporating structural and geometric characteristics of the gel matrix, M_t is the amout of naringin released at time t, M_∞ is the amout of naringin released at infinite time and n is the release exponent indicative of release mechanism. The release pattern was primarily influenced by the proportion of gellan gum as observed from Figure S2. The formulations formed thick gel in presence of SNF and prolonged the release. The rapidity in gelation avoided burst release and also

extended the release. Non-Fickian anomalous (*n* > 0.45) transport based release mechanism. Quick gelation in presence of SNF avoided the possibility of free diffusion of emulsion droplets.

S4. FT-IR spectra

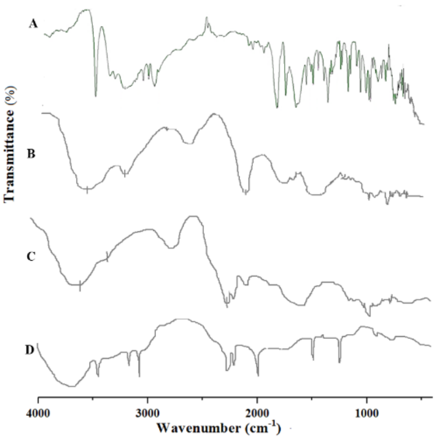

Figure S3. FT-IR spectra of A: Naringin, B: Gellan gum C: Gellan gum after gelation D: Naringin loaded in situ gelling nanoemulgel.

The molecular characterization of naringin and its nanoemulgel was carried out at room temperature using FTIR spectrometer (Bruker, Ettlingen, Germany) equipped with an attenuated total reflection (ATR) accessories. All measurements were performed between 4000 and 400 cm^{-1}. The FTIR spectra of naringin and gellan gum was recorded in powder mode after mixing with potassium bromide in an agate mortar, and pressed into pellets for recording the IR spectra. For gelled gellan gum and naringin loaded in situ gelling nanoemulgel ATR was used to obtain the FTIR spectra with a resolution of 4 cm^{-1}. Naringin has shown characteristic peaks in the FT-IR spectrum (Figure S3A) characterised by vibrational bands due to O-H, C-H, C=O, and C-H functional groups at 3463, 2989, 1656, and 2989 cm^{-1} respectively. In case of gellan gum (Figure S3B), the characteristic peaks were observed due to stretch vibration of O–H and C–H at 3425 $cm^{-1,}$ and 3193 cm^{-1}. Stretching of asymmetric carboxylate anion, symmetric carboxylate anion and C–O is observed at 1700, 1420, and 989 cm^{-1} respectively. After gelation, due to the ionic interaction between carboxylic groups of gellan gum and the cations (Figure S3C), shift in the bands of symmetric carboxylate anion stretching and C–O stretching was observed shifted to 1509 and 1027 cm^{-1}, respectively.

S5. HPLC method validation

Table S3. RP-HPLC method validation parameters for the estimation of naringin.

Method parameter	Results
Injection precision	Relative standrad deviation 0.39 (*n* = 6)
Tailing factor	1.04 ± 0.02
Theoretical plates	9560 ± 135
Linearity	0.05–50 µg/mL
Accuracy (%)	99.3 ± 0.19 (Mean % recovery at 3 different spiked levels)
LOD and LOQ	15 ng/mL and 50 ng/mL
